# Social context and dominance status contribute to sleep patterns and quality in groups of freely-moving mice

**DOI:** 10.1038/s41598-019-51375-7

**Published:** 2019-10-23

**Authors:** Stoyo Karamihalev, Cornelia Flachskamm, Noa Eren, Mayumi Kimura, Alon Chen

**Affiliations:** 1 Department of Stress Neurobiology and Neurogenetics, Max Planck Institute of Psychiatry, Munich, 80804 Germany; 2 Department of Neurobiology, Weizmann Institute of Science, Rehovot, 76100 Israel; 3 International Research Center for Neurointelligence (WPI-IRCN), The University of Tokyo Institute for Advanced Study, Tokyo, Japan

**Keywords:** Computational neuroscience, Social behaviour

## Abstract

In socially-living species, sleep patterns are often subject to group influences, as individuals adjust to the presence, daily rhythms, and social pressures of cohabitation. However, sleep studies in mice are typically conducted in single-housed individuals. Here, we investigated sleep in a semi-naturalistic environment with freely-moving, group-housed mice using wireless electroencephalographic (EEG) monitoring and video tracking. We found evidence of in-group synchrony of sleep state patterns and effects of social dominance status on sleep quality. These findings highlight the importance of exploring sleep in a social context and are a step toward more informative research on the interplay between social functioning and sleep.

## Introduction

Disturbances in social functioning are comorbid with sleep problems in many prevalent psychiatric disorders, most notably autism-spectrum, mood, and anxiety disorders^1–3^. Our understanding of the common causality and the interplay between sleep impairment and psychiatric symptomatology could greatly benefit from experimental paradigms that allow simultaneous assessment of both domains of functioning. In rodents, much is known about the sensitivity of sleep structure and quality to a variety of experimental manipulations. However, despite the advent of telemetric systems allowing for untethered EEG recordings^4,5^, most studies to date were performed on single-housed animals, neglecting the influence of social dynamics and group-derived individual differences.

In the few studies that have examined the effect of the social environment on sleep, it was shown that social isolation blunts the homeostatic response to sleep deprivation compared to pair-housed conditions^6^, and that the number and duration of rapid eye movement (REM) sleep bouts differ between single and group-housed male mice^7^. An additional study demonstrated that synchronization of the circadian rhythmicity of body temperature in female mice depends on the number of individuals in a group^8^. These findings emphasize the need to consider social context in studies of sleep and circadian outcomes. Importantly, however, the role of the individual within the group was not addressed.

In this work, we investigated the effects of group social dynamics on sleep in male mice. Both sleep and behavioral measurements were obtained over several days from group-housed mice living in an enriched, semi-naturalistic environment. We show that group living affects the sleep-wake dynamics of the individuals in the group, and that dominance status influences specific sleep characteristics, an effect that may be modulated by acute stress.

## Results

**Figure 1:**
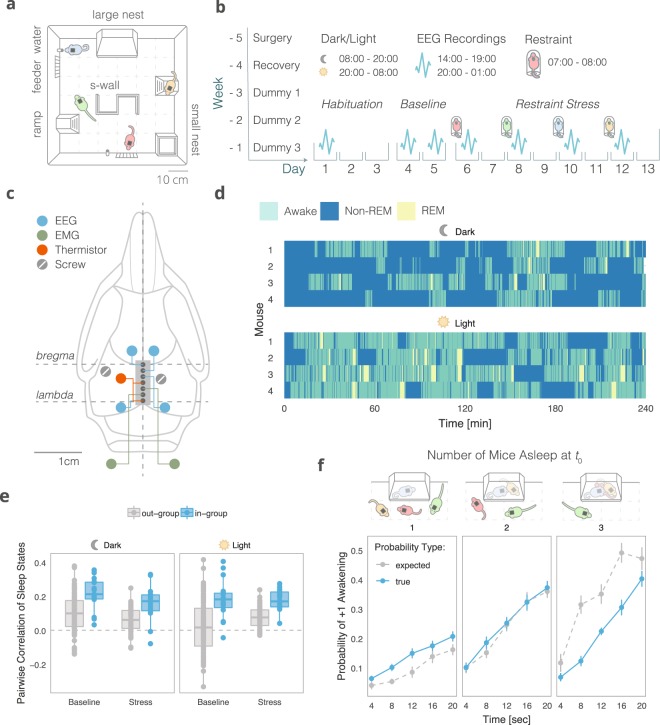
Sleep coordination in freely moving groups of mice. (**a**) The “Social Box” (SB) paradigm (described in detail in refs^9–11^). Each SB is an arena where a group of individuals cohabitate under continuous video monitoring for the duration of the experiment (several days). Each box contains a closed large nest, a small open shelter, two ramps, two feeders, two water bottles, and an S-wall. **(b)** Experimental timeline. Implantation of EEG and EMG electrodes was performed five weeks before introduction to the SB. The surgery was followed by a week of recovery and three training weeks of habituating the animals to dummy head stages of increasing weights. Baseline behavioral and EEG recordings were collected for 5 days, followed by individual stress (1 h restraint) before the beginning of the dark phase. A different animal from the same group was stressed every other day. **(c)** The coordinates of each recording electrode. Polygraphic signals were collected from four EEG channels, two EMG channels, and a thermistor. **(d)** Representative hypnograms from a single group of four mice (dark and light phase recordings, 14:00–19:00 & 20:00–01:00, resp.). **(e)** Pairwise correlations of sleep states. In-group sleep state correlations are higher than out-group correlations, indicating in-group sleep synchrony (in-group vs. out-group, box-plot: line – median, box limits – 1^st^ and 3^rd^ quartile, whiskers – 1.5 × IQR, *n* = 120 pairs). **(f)** Push-pull group effects on sleep. The probability of a mouse awakening within 5 epochs (20 seconds) if it is the only mouse in a group asleep at *t*_0_ is higher than expected based on out-group measurements. Conversely, with three mice asleep at *t*_0_, the probability of one of them awakening in the same time frame is significantly lower than expected by chance (interaction of group type and number of mice, mean ± SE, *n* = 16 individuals).

In order to study behavior in group-living mice, we used the “Social Box” (SB) paradigm, wherein a group of mice live together in an enriched environment under continuous video observation (Fig. 1a; Movie 1)^9,10^. Mice were assigned to groups of four non-littermates from weaning and remained in these groups until testing in adulthood (>10 weeks old). An automatic tracking system recorded the movement of each mouse in the SB, allowing us to detect and quantify the numbers, directionalities, and types of social interactions they displayed (*see Methods*). Wireless EEG and electromyographic (EMG) recordings were acquired from each mouse in the group simultaneously at several time-points, including both dark phase and light phase recordings (the first 4 h of high-quality data collected in a 5 h recording period; Figs 1b,c and S2).

The ability to simultaneously collect sleep data from multiple group-housed individuals gave us the unique opportunity to investigate in-group temporal sleep synchrony (Fig. 1d). To quantify sleep state synchrony, we dichotomized the scored sleep data into awake or asleep (both REM and NREM) and calculated the pairwise correlations in sleep states between animals over time (Pearson’s correlation coefficient of two binary vectors). To account for predictable circadian sleep patterns that occur innately in mice, independent of group belonging, we compared pairwise correlations for mice belonging to the same group (in-group) against out-group correlations. During both the dark and light phases, baseline in-group correlations of sleep states were significantly higher than out-group correlations (permutation test, *Sum Sq*.(1, 116) = 0.723, *p* = 2.2 × 10^−16^, *iterations* = 5000), suggesting inherent coherence in the group’s sleep dynamic (Fig. 1e). The extent of these correlations does not appear to change during the stress phase of the experiment (following 1 h of acute restraint stress, Fig. 1b) in a group type-specific manner (permutation test − group type × stage interaction, *Sum Sq*. (1, 116) = 0.0239, *p* = 0.1366, *iterations* = 647).

To further explore sleep cohesion at baseline, we calculated the average probability of one or more additional animals waking up within 5 epochs (20 seconds) of an individual’s awakening at time-point zero (*t*_0_, Fig. 1f). We accounted for group influences by conditioning these probabilities on the number of individuals asleep at *t*_0_. We found that if only one mouse was asleep in a group at a given time, the true probability of it waking up within the allotted time interval is significantly higher than the expected probability (based on a comparison against out-group predictions). Conversely, if only one individual is awake at *t*_0_, the true probability of one of the three other individuals waking up is significantly reduced (probability type × N mice, permutation test with time-point as repeated measure, *Sum Sq*.(4, 357) = 0.333, *p* < 2 × 10^−16^). When half of the group is asleep at *t*_0_, the true and expected probabilities of an additional awakening overlap. These results suggest that social context has a push-pull effect on an individual’s sleep pattern, wherein a mouse is more likely to wake up if its conspecifics are awake, and more likely to stay asleep if its conspecifics are asleep.

Social dominance status was assessed based on the numbers and directionality of aggressive interactions displayed during the first 12 h dark-phase recording. A dominance score (David’s Score, *see Methods*) was assigned to each mouse^9,11^. Additionally, ranking this score allowed for the separation of individuals in each group into dominant (DOM, ranks alpha and beta) and subordinate (SUB, ranks gamma and delta). This binary split was created to give equal sample sizes for each dominance status without segregating the individuals into too many groups, however we additionally looked at correlations with the David’s Score directly. The assignment of dominance rankings based on the first night, rather than the entire monitoring period, made it possible for us to draw conclusions about the predictive power of social rank.

**Figure 2:**
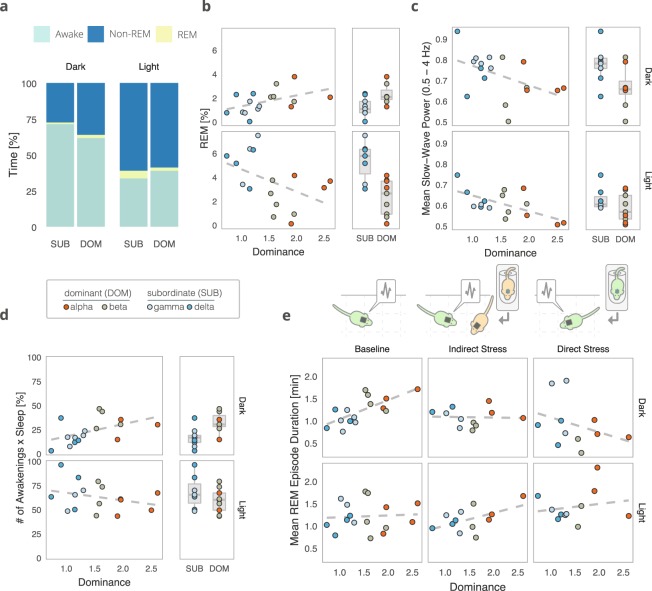
Social dominance status predicts sleep characteristics. (**a)** Average percent of time spent in each sleep stage differs between subordinate (SUB, ranks gamma and delta) and dominant (DOM, ranks alpha and beta) animals, adjusted for between-group differences (approx. 4 h of recording time per individual per light phase, *n* = 18 individuals). **(b)** Baseline dominance (David’s Score rank) predicts increased group-adjusted dark phase REM sleep and decreased light phase REM sleep (*n* = 18 individuals). **(c)** Lower-ranking individuals showed higher slow-wave activity during NREM sleep, suggesting baseline dominance levels predict group-adjusted mean NREM slow-wave power (*n* = 18 individuals). **(d)** Sleep fragmentation, corrected for total sleep amount and group belonging, is increased in dominant animals compared to subordinates during the dark phase (*n* = 18 individuals). **(e)** Social dominance mediates the effects of stress on group-adjusted mean REM episode duration (*n* = 18 individuals). For all panels, box-plot elements are as follows: line – median, box limits – 1^st^ and 3^rd^ quartile, whiskers – 1.5 × IQR.

The average sleep state proportions for SUB and DOM individuals during both the light and dark phase recordings are summarized in Fig. 2a. Curiously, while dominance contributed significantly to the proportion of REM sleep both in the dark and light phases, the association was positive in the dark phase and negative in the light phase (Fig. 2b, rank × light phase interaction, *F*(1, 12) = 24.971, *p* = 0.0003). We speculate that the decrease in REM sleep seen in the dark phase for SUB individuals could be interpreted as the acute suppression of REM following the stress associated with negative social interactions during the dark phase^12^. This may be followed by REM rebound during the light phase.

Slow-wave (0.5–4 Hz) power during NREM sleep reflects sleep intensity and homeostasis^13^. We found that the mean slow-wave power during NREM sleep differs along dominance ranks, such that socially dominant individuals had overall reduced slow-wave activity (*F*(1, 12) = 5.576, *p* = 0.036), an effect which was especially pronounced during the light phase. Concordant with this, we found sleep fragmentation to be higher in DOM individuals during the dark phase (Fig. 2d, *F*(1, 10) = 7.368, *p* = 0.0218 for post-hoc comparison prompted by a significant interaction effect of rank and light phase on sleep fragmentation, *F*(1, 12) = 7.57, *p* = 0.0175).

To test if the relationship between social dominance and sleep properties is sensitive to psychological stress, we compared sleep characteristics across social ranks at baseline with direct stress (1 h restraint, performed at the end of the light phase) or indirect stress (another individual in the same group exposed to 1 h of restraint). A strong relationship between dominance and mean REM episode duration, present at baseline in the dark phase, was abolished if a stressed individual was present in the group, and reversed under direct stress (Fig. 2e, dominance × mean REM episode duration in the dark phase, *F*(2, 16) = 6.032, *p* = 0.0112). These findings suggest that the association between dominance and sleep physiology may be disturbed by exposure to acute stress.

## Discussion

Social cohabitation is beneficial to mice and humans, offering, among other advantages, protection from predation and opportunities for sharing of resources and parental care. These benefits, however, come at the expense of an individual having to adjust to group norms and compete with other group members for resources.

In this work we have shown that social cohabitation sets the rhythm of sleep-wake behavior for group-living male mice. Given that a certain level of sleep synchronization seems to be the natural dynamic of a group, these findings suggest the possibility of psychiatric symptomatology manifesting as disturbances in adjustment to the sleep states of others. Indeed, the severity of insomnia in autistic adults was found to be positively associated with lower social skills^14^. We hypothesize that, for example, mouse models of autism spectrum disorder would show impairment in sensitivity to the sleep-wake patterns of their group-members. While a few studies have already demonstrated sleep impairments in autistic-like rodents^15–17^, the effect of the social environment on sleep has yet to be investigated in autism spectrum models.

Moreover, while cohabitation creates synchrony in overall sleep patterns, we have shown that group dynamics can also intensify individual differences. Social dominance is among the most prominent and differentiating individual characteristic in male mice^11^. Here we show social rank associations with differences in sleep architecture, REM episode duration, and slow-wave activity during NREM sleep. While little is known about the effects of social dominance on sleep in rodents, chronic subordination stress has been studied using the social defeat paradigm. Social defeat is a potent social stressor with etiological and face validity to several stress-related disorders in humans^18,19^ and has been utilized in a number of sleep studies to date. Acute social defeat was shown to produce an increase in NREM sleep duration and slow-wave activity in both rats and mice, as well an immediate reduction in REM sleep duration, followed by a rebound, in mice only^12,20,21^. Our finding of increased dark phase slow-wave activity in combination with a pronounced increase in light phase REM sleep in subordinate mice may reflect a response to aggressive social interactions during the preceding dark phase, which likely carry some similarity to episodes of acute social defeat for the subordinate animals. It should be noted, however, that a study in rats has shown both winners and losers of an acute aggressive encounter can respond in a similar way^22^.

Additionally, chronic (10-day) social defeat paradigms elicit similar changes in sleep architecture and power spectra during the protocol, but outcomes in the recovery period differ between susceptible and resilient mice. Changes in sleep architecture and circadian rhythmicity of activity and body temperature appear to persist only in susceptible mice during recovery^23,24^. These findings indicate that there may be important pre-existing individual differences contributing to the susceptibility to sleep disturbances upon stress. We hypothesize that one such pre-existing characteristic may be social dominance status. While we cannot conclude based on our current work that susceptibility to sleep abnormalities upon chronic stress is mediated by social dominance status, this represents an intriguing hypothesis to pursue in the future.

Finally, we have shown that the relationship between dominance and REM bout duration during the dark phase is sensitive to both direct and indirect stress. The social transmission of stress between cohabitating animals has been previously demonstrated^25,26^, and quite a few studies have looked into the effects of acute restraint stress on sleep in rodents (reviewed in ref.^27^). Our results indicate that the individual’s characteristics and social environment may account for some of the variation seen in such studies.

It is important to note that the methods used in this study have several key limitations. The battery life of our wireless telemetric devices limited recording duration to five hours, with the signal deteriorating in quality during the last hour, forcing us to keep only four hours of signal per recording. This is much shorter than typically seen in sleep studies, where EEG signal is conventionally collected continuously for multiple days. We have tried to compensate for this issue by performing multiple recording sessions per individual. This, however, has the downside that each mouse was handled by an experimenter multiple times, as the real transmitter and battery had to be installed prior to each recording.

In conclusion, simultaneous assessment of sleep and social behavior has allowed us to attempt a relatively detailed exploration of the connection between two separate, yet very much intertwined domains of neurobiological functioning. We believe that this work emphasizes the importance of exploring sleep in a social environment and offers a way toward improved animal models of psychiatric disorders of sleep and social functioning.

## Methods

### Animal housing and care

Male CD-1 (ICR) mice were bred and housed in an SPF-facility in temperature-controlled rooms under a 12 h light/dark cycle with food and water available *ad libitum*. Upon weaning, mice were transferred into groups of four non-littermates and housed together until adulthood (>10 weeks of age). All animal studies were carried out in accordance with the European Community Council Directive. Animal experimental protocols were approved by the local commission for the Care and Use of Laboratory Animals of the Government of Upper Bavaria, Germany. All mice were marked prior to the experiments to enable automatic video color tracking. Fur coloring using hair dyes (Tish & Snooky’s NYC Inc., New York) under mild isoflurane anesthesia was performed as described elsewhere^9,11^.

### Wireless telemetry system

The wireless transmitters were custom-made by Multi Channel Systems GmbH (Reutlingen, Germany, S1 A-B). Each transmitter weighed ca. 3 g and was attached to a seven-pin connector (Preci-Dip Durtal SA, Delémont, Switzerland). A detachable 100 mAh battery, weighing ca. 4 g, provided a maximum of five hours of continuous recording time at a sampling rate of 1 kHz (S2). A receiver (Wireless 2100-RE, Multi Channel Systems GmbH, Reutlingen, Germany) was placed in a corner on top of the SB frame, such that any position in the social box was less than 1 m away. The receiver was connected to a computer via an interface board (Wireless 2100-IFB). Dedicated Multi Channel Systems software was used for acquisition (Multi Channel Experimenter, v. 1.0.0.1, Reutlingen, Germany). An electrode implant unit, consisting of a central seven-pin connector, was equipped with four soldered gold wire EEG electrodes, two gold wire EMG electrodes, and a thermistor (Tewa Temperature Sensors, Lublin, Poland), as described previously^28^.

### Surgical procedures

Surgery was performed under inhalation anesthesia (mixture of isoflurane and oxygen) in a stereotaxic frame. Two EEG electrodes were inserted bilaterally anterior to Bregma (+1.5 mm AP, ± 1 mm ML) and two more were inserted over the parietal cortex (AP −1 mm, ML ± 3 mm, Fig. 1c). The thermistor was implanted unilaterally (AP −2 mm, ML 2 mm) for brain temperature monitoring. Additionally, two EMG electrodes were implanted into the trapezoid muscles. Finally, two anchor screws were placed into the skull for improved stability of the implant. The complete unit and screws were fixed to the cranial bone with dental resin. The entire surgical procedure lasted approximately 25 min per mouse. Prior to surgery, each animal received a subcutaneous injection of atropine sulfate (0.05 mg/kg, Atropine, Braun Melsungen, Melsungen, Germany) for stabilization of cardiovascular function and meloxicam (1 mg/kg, Metacam, Braun Melsungen, Melsungen, Germany) for analgesia. Meloxicam was additionally administered at 24 h and 48 h after surgery.

The surgery was followed by a week of recovery, during which time animals were housed in their original groups and bodyweights were monitored daily. For three weeks after the recovery week, the animals were acclimated to carry progressively heavier dummy-transmitter devices (custom-made using aluminum plates in the shape of the transmitter, dummy 1–2 g, dummy 2 – ca. 4 g, and dummy 3 – ca. 7 g). Each dummy was worn continuously for a week in the three weeks leading up to the first measurement (Fig. 1b, Movie 1). During the entire three-week training period, the animals were habituated to gentle daily handling.

### Data acquisition and timeline

At the start of each recording, each mouse was removed from the SB for several minutes. During this time, the dummy transmitter was replaced with a real transmitter with a charged battery attached. The mouse was then reintroduced into the SB. The transmitter and battery were replaced with the 4 g dummy the following day, which was worn until the next recording. Recordings were collected between 14:00 and 19:00 and between 20:00 and 01:00 on days 1, 4, 5, 6, 8, 10, and 12 of the SB observation period (Fig. 1b). “Baseline” recordings were acquired after a period of three days during which the mice became accustomed to the social box environment. “Stress” recordings were conducted one day apart on days 6, 8, 10, and 12 following an hour of restraint stress (07:00–08:00, Fig. 1b) performed outside of the SB. Each individual in each group was exposed to restraint once on only one of these four days. The order of stress exposure within the group was randomized. The individual that underwent restraint was considered directly stressed, while the other three individuals were labeled as indirectly stressed, as they were exposed to a mouse that had been stressed directly.

### Telemetric data processing and sleep-wake classification

Telemetric data was processed offline for analysis with a LabVIEW-based acquisition program (National Instruments, Austin, TX, USA), customized for use in mice. EEG and EMG signals were amplified 10000 times and filtered (EEG: 0.1–460 Hz, EMG: >200 Hz).

Sleep/wake states were determined manually by an experienced scorer simultaneously considering the parietal EEG as well as the EMG signal. Vigilance states were characterized as “awake”, “REM”, and “non-REM” (non-rapid eye movement sleep).

A Fast Fourier Transform (FFT) algorithm was used on data binned into four-second epochs for power spectral analysis. Mean values of the EEG spectrum/0.25 Hz were calculated and normalized per animal per recording using the individual mean of the total EEG power from all vigilance states across all frequency bins and epochs. Slow-wave activity during NREM sleep was assessed by summing over the power densities between 0.5 Hz and 4 Hz. This power was then averaged per animal across the measurement period.

### The social box and automated behavioral tracking

The SB (Fig. 1a) is an enriched housing environment designed to house groups of mice as described in detail elsewhere^9–11^. The behavior of mice was recorded continuously and tracked automatically using a specialized software written in Matlab (Mathworks Inc.).

### Assignment of dominance ranks

Agonistic interactions between mice throughout the SB monitoring period were captured and classified based on movement trajectories as described in detail elsewhere^9,11^. The David’s Score was used as a continuous in-group measure of dominance, calculated based on the numbers and directionalities of aggressive chases^11,29^. A detailed description of how the David’s Score is calculated is provided elsewhere^11^. The score assumes a linear hierarchy in a group and is normalized within-group to between 0 and 3 (*n-1* mice in a group, higher means more dominant).

To create discrete groups, mice were additionally ranked based on this score and the two top-ranking individuals were considered to be dominant (“DOM”) while the 3^rd^ and 4^th^ ranking individuals were considered subordinate (“SUB”).

### Statistical analyses

All statistical analyses were performed in R (www.R-project.org). Mixed effects modelling was aided by the “nlme” package^30^. Permutation-based testing was performed using the “lmPerm” package^31^. All dependent variables were tested for normality and homoscedasticity. Permutation-based or other non-parametric alternatives were employed whenever these assumptions were not met.

Pairwise correlations between sleep states (Fig. 1e) were assessed for all pairs of mice included in the experiment, independent of group belonging, using the Pearson product moment correlation coefficient. Subsequently, correlation values derived from pairs of individuals belonging to the same group were compared to those of between-group pairs. While the recordings for different groups were not collected simultaneously, all recordings were collected at the same times during the day across groups and thus capture the same circadian segments. This allowed us to use between-group correlations as a reasonable control for expected synchrony in sleep states independent of group belonging.

All tests of social dominance against sleep properties included group belonging as a covariate factor in the analysis, thus adjusting for the effects of group differences (individual mouse nested within group). Accordingly, the values plotted for such comparisons are always adjusted for group effects.

All the code for performing the analyses and generating the figures for this manuscript, as well as multiple additional details regarding the statistical properties of experimental samples and readouts are available in an online repository (https://stoyokaramihalev.github.io/EEG_sleep).

## Supplementary information

Supplementary Info

Movie 1

## Data Availability

All data used to support the findings of this work are available from the corresponding author upon reasonable request.

## Code availability

The code used in performing the analyses and producing the figures for this manuscript is freely accessible in a GitHub repository: https://stoyokaramihalev.github.io/EEG_sleep. The MATLAB-based mouse tracking system is available from the corresponding author upon request.
