# Supplementary material for: Social context and dominance status contribute to sleep patterns and quality in groups of freely-moving mice
Source: Sci Rep. 2019 Oct 23;9:15190. doi: 10.1038/s41598-019-51375-7 (PMC6811636; doi:10.1038/s41598-019-51375-7)
Supplement: Supplementary file 1 — Supplementary Info [file 41598_2019_51375_MOESM1_ESM.pdf]

Title

Social context and dominance status contribute to sleep patterns and quality in groups of freely-moving mice

Author list and affiliations

Stoyo Karamihalev[1], Cornelia Flachskamm[1], Noa Eren[2], Mayumi Kimura[3], and Alon Chen[1,2]*

[1]Department of Stress Neurobiology and Neurogenetics, Max Planck Institute of Psychiatry, Munich, 80804, Germany.

[2]Department of Neurobiology, Weizmann Institute of Science, Rehovot, 76100, Israel.

[3]International Research Center for Neurointelligence (WPI-IRCN), The University of Tokyo Institute for Advanced Study, Tokyo, Japan

*Corresponding author: Alon Chen, Department of Stress Neurobiology and Neurogenetics, Max Planck Institute of Psychiatry, Munich, 80804, Germany. +49 (0) 89-30622-586; alon_chen@psych.mpg.de

Supplementary Information

Movie 1. A representative clip from a Social Box recording. Demonstration recording of four mice, implanted with EEG transmitters, moving freely inside the behavioral arena.
